# Unraveling the Connection: Pain and Endoplasmic Reticulum Stress

**DOI:** 10.3390/ijms25094995

**Published:** 2024-05-03

**Authors:** Ryoko Kawanaka, Hisayo Jin, Tomohiko Aoe

**Affiliations:** 1 Department of Anesthesiology, Chiba Medical Center, Teikyo University, Ichihara 299-0111, Japan; 2 Department of Anesthesiology, Chiba University Graduate School of Medicine, Chiba 260-8670, Japan; 3 Pain Center, Chiba Medical Center, Teikyo University, Ichihara 299-0111, Japan

**Keywords:** pain, ER stress, UPR, chaperone, protein quality control

## Abstract

Pain is a complex and multifaceted experience. Recent research has increasingly focused on the role of endoplasmic reticulum (ER) stress in the induction and modulation of pain. The ER is an essential organelle for cells and plays a key role in protein folding and calcium dynamics. Various pathological conditions, such as ischemia, hypoxia, toxic substances, and increased protein production, may disturb protein folding, causing an increase in misfolding proteins in the ER. Such an overload of the folding process leads to ER stress and causes the unfolded protein response (UPR), which increases folding capacity in the ER. Uncompensated ER stress impairs intracellular signaling and cell function, resulting in various diseases, such as diabetes and degenerative neurological diseases. ER stress may be a critical universal mechanism underlying human diseases. Pain sensations involve the central as well as peripheral nervous systems. Several preclinical studies indicate that ER stress in the nervous system is enhanced in various painful states, especially in neuropathic pain conditions. The purpose of this narrative review is to uncover the intricate relationship between ER stress and pain, exploring molecular pathways, implications for various pain conditions, and potential therapeutic strategies.

## 1. Introduction

Mature proteins whose higher-order structures have been properly formed through protein quality control are transported to appropriate locations within cells [1]. The impairment of this process triggers an integrated stress response not only in the cytoplasm but also in the endoplasmic reticulum (ER) [2,3]. The unfolded protein response (UPR) compensates for the overload of misfolding proteins in the ER caused by insults such as oxidative stress, aging, malnutrition, and gene modification [4]. The stress response affects various cellular activities through altered intracellular signaling and is often associated with pathogenic conditions [5]. In addition, if the stress is not compensated for, cellular dysfunction and cell death occur, causing various disease conditions such as neurodegenerative diseases [6], diabetes [7], and myocardial disorders [8]. Individuals are composed of various organs and cells, and disruption of the ER function in certain cell types impairs the functions performed by these cells and organs. If the cells are beta cells in the pancreas, the resulting disease is diabetes [9], whereas if the cells are dopamine-producing nerve cells, the resulting disease is Parkinson’s disease [10]. Similarly, ER-stress-related damage [11,12] and UPR-induced changes in intracellular signaling [13,14,15] in sensory nervous systems may influence acute and chronic pain. In this review, we consider whether universal cellular responses of ER stress and the UPR may influence pain pathology like for other diseases.

## 2. Type of Pain

Pain is a subjective experience, characterized by “an unpleasant sensory and emotional experience associated with, or resembling that associated with, actual or potential tissue damage” (International Association for the Study of Pain (IASP), https://www.iasp-pain.org/resources/terminology/, accessed on 27 January 2024). Pain has a crucial biological function: signaling the presence of harm or potential harm to the body and initiating appropriate behavioral and physiological responses. Nociceptive, neuropathic, and nociplastic pain are three distinct categories of pain, each with unique characteristics, underlying mechanisms, and physiological implications [16].

### 2.1. Nociceptive Pain

Nociceptive pain results from the activation of sensory neurons, known as nociceptors, which respond to potentially damaging stimuli such as tissue injury and extreme temperatures [17]. This type of pain occurs in response to various physical and chemical agents like trauma, surgery, and chemical burns. The process of nociception involves the central and peripheral nervous systems. When noxious stimuli occur, such as tissue damage or exposure to extreme temperatures, nociceptors are activated. There are two main types of nociceptive nerve fibers: Aδ fibers and C fibers [18]. Aδ fibers have a limited receptive field that allows the body to localize pain. These fibers are myelinated and are responsible for the early perception of pain. In contrast, C fibers are unmyelinated and have a wide receptive field, allowing them to convey the intensity of pain [19]. These neurons then convey signals to the central nervous system. The perception of pain depends on several factors, including the frequency of action potentials, the intervals between action potentials, and input from higher-order brain centers [18]. In cases of persistent noxious stimuli, nociceptive neurons can release proinflammatory cytokines, leading to inflammation in the local environment. This response can sensitize or activate nearby cells, leading to a spread of inflammation beyond the initial area of nociceptor activation—a phenomenon known as neurogenic inflammation [20]. Nociceptive signals cease once the stimulus ends, the receptors are suppressed, or an influx of calcium causes the nerve endings to collapse and become refractory to further stimulation. Nonsteroidal anti-inflammatory drugs (NSAIDs), acetaminophen, and opioids are used to treat nociceptive pain [21].

### 2.2. Neuropathic Pain

Neuropathic pain is a common chronic pain disorder caused by lesions or diseases of the somatosensory nervous system, including trigeminal neuralgia, painful polyneuropathy, postherpetic neuralgia, and central post-stroke pain [22]. This system includes peripheral nerves and central neurons. Approximately 7–8% of the general population is affected by neuropathic pain with continuous or intermittent spontaneous pain, including burning, pricking, or squeezing [22,23]. This pain can sometimes be triggered in response to thermal stimulation or slight touch. The mechanisms underlying neuropathic pain include the abnormal ectopic activity of nociceptive nerves, peripheral and central sensitization, impaired inhibitory regulation, and pathological activation of the microglia [22,24]. Treatment approaches for neuropathic pain include nonpharmacological, pharmacological, and interventional therapies, with the most substantial evidence available for pharmacological treatments [24]. Therapeutic drugs include anticonvulsants, which act on the calcium channel α2δ subunit (such as gabapentin and pregabalin); lidocaine, which acts on sodium channels; and antidepressants, which act on the descending regulatory inhibition pathway (such as tricyclics and serotonin–norepinephrine reuptake inhibitors) [22,24].

### 2.3. Nociplastic Pain

Nociplastic pain is a relatively recent concept in pain classification, in addition to neuropathic pain and nociceptive pain. The condition is characterized by the lack of a direct link between the pain experienced and any identifiable physical or neurological damage [25]. The pathophysiology of nociceptive pain is not completely understood but is thought to involve changes in the way the nervous system processes pain signals [25]. Unlike nociceptive pain, which results from actual or potential tissue damage, and neuropathic pain, which results from direct nerve damage, nociplastic pain represents a change in the way pain is perceived [26]. Nociplastic pain, which is associated with conditions such as fibromyalgia, involves impaired central nervous system pain processing [27]. This type of pain may not respond well to traditional pain medications or physical interventions. Treatment often focuses on multidisciplinary approaches, including physical therapy, psychological support, and certain medications [25].

It is necessary to understand these differences in pain and use effective treatments depending on the type of pain.

## 3. The ER and the UPR

**Figure 1:**
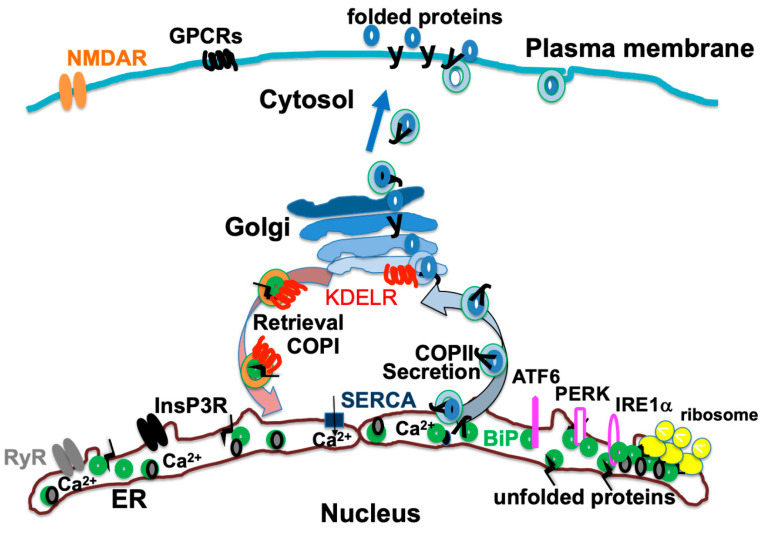
Nascent proteins are inserted into the endoplasmic reticulum (ER) and their folding is facilitated by the interaction with ER chaperones such as binding immunoglobulin protein (BiP). Mature proteins are then secreted by coat protein II (COPII)-mediated vesicular transport. Some ER-resident chaperones like BiP are also secreted in this way, but their KDEL sequences are recognized by the KDEL receptor (KDELR), which results in the chaperones being transported from the Golgi back to the ER by coat protein I (COPI) vesicular transport. NMDAR, N-methyl-D-aspartate receptor; GPCRs, G protein-coupled receptors; SERCA, sarcoplasmic reticulum calcium ATPase; InsP3R, inositol 1,4,5-trisphosphate receptor; RyR, ryanodine receptor; ATF6, activating transcription factor 6; PERK, protein kinase R (PKR)-like ER kinase; IRE1α, inositol-requiring enzyme 1α. Figure 1 is reproduced from the figures in Aoe, T. Pathological Aspects of COVID-19 as a Conformational Disease and the Use of Pharmacological Chaperones as a Potential Therapeutic Strategy. *Front Pharmacol* 2020, 11, 1095 (doi:10.3389/fphar.2020.01095) [29].

The ER plays an important role in a variety of cellular functions that are essential for the healthy functioning of an organism, such as protein folding and Ca^2+^ signaling [28] (Figure 1).

### 3.1. Protein Folding

Newly synthesized polypeptides on ribosomes in the ER membrane are inserted into the ER lumen from the Sec61 translocon [1], and secretory proteins, membrane proteins, and organelle proteins undergo folding in the ER [30]. These nascent polypeptides bind to ER molecular chaperones, such as immunoglobulin heavy chain binding protein (BiP/GRP78), calreticulin, and protein disulfide isomerase (PDI), present in the ER lumen and calnexin present in the ER membrane, which prevents aggregation and degradation of the polypeptides [31]. The polypeptides also undergo modifications such as signal peptide removal, N-linked glycosylation, and the formation of disulfide bonds [32]. Protein complexes consisting of several subunits are formed. Molecular chaperones in the ER help overcome the physicochemical and cell biological constraints of folding, and facilitate compatibility with subsequent intracellular transport [32].

Mature proteins with proper tertiary structures are separated from the ER chaperones, secreted from the ER to the Golgi, and transported to other cellular compartments, such as endosomes and the plasma membrane, or secreted from the cell [30]. The ER maintains homeostasis despite the influx of newly synthesized proteins. Proper protein folding and quality control in the ER are essential for cellular protein homeostasis and preventing the accumulation of defective proteins [33]. The complex mechanism of protein folding in the ER and its impact on various diseases are gradually being elucidated [5,34]. Perturbations in ER homeostasis can lead to the misfolding of proteins, triggering ER stress and the UPR [2].

### 3.2. Ca^2+^ Signaling

The ER is the major organelle for the storage of intracellular Ca^2+^ and controls calcium dynamics [28]. This regulation is essential for creating a favorable environment for protein folding and for serving as a reservoir for the rapid and specific release of calcium [35]. Agonist-induced fluctuations in the concentration of Ca^2+^ within the ER can affect many ER functions, including protein synthesis, protein modifications, and chaperone–chaperone interactions [36]. The binding of a ligand to a G protein-coupled receptor (GPCR) on the cell surface, for example, the μ-opioid receptor (MOR) and metabotropic glutamate receptors, activates phospholipase C, which mediates inositol 1,4,5-trisphosphate (InsP3) and diacylglycerol production from phosphatidylinositol 4,5-bisphosphate. InsP3 causes Ca^2+^ release into the cytoplasm via InsP3 receptors on the ER membrane [37,38]. Another Ca^2+^ channel is ryanodine receptors [39]. Ca^2+^ flows into the cell via ion channels such as ionotropic glutamate receptors present on the plasma membrane [40]. The increased cytoplasmic concentration of Ca^2+^ promotes further Ca^2+^ release from the ER to the cytoplasm. This calcium-induced calcium release (CICR) occurs via ryanodine receptors present on the ER membrane, which was first discovered in skeletal muscle [41]. Three subtypes of ryanodine receptor gene exist, and all three are expressed in the brain [42]. CICR occurs in hippocampal neurons through an increase in the concentration of intracellular Ca^2+^ due to extracellular Ca^2+^ inflow through N-methyl-D-aspartate (NMDA)-type glutamate receptors [43].

**Figure 2:**
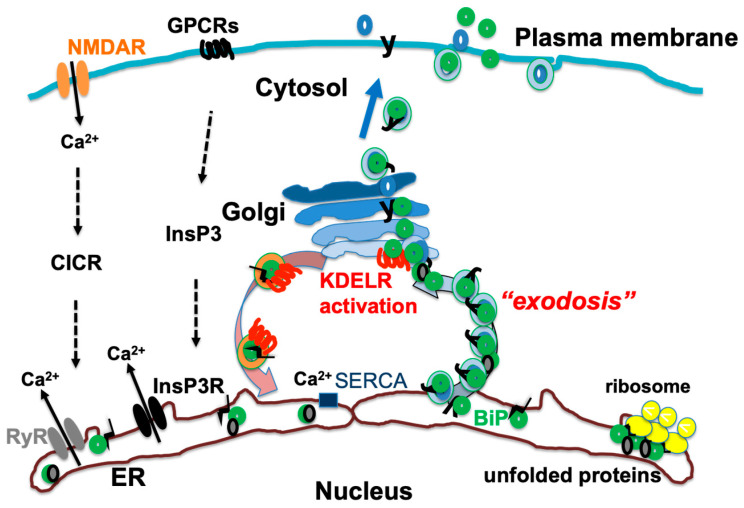
Activation of N-methyl-D-aspartate receptor (NMDAR) and G protein-coupled receptors (GPCRs) leads to release of Ca^2+^ from endoplasmic reticulum (ER) through ryanodine receptor (RyR) and inositol 1,4,5-trisphosphate receptor (InsP3R). In addition, Ca^2+^ is recovered from cytosol to ER by sarcoplasmic reticulum calcium ATPase (SERCA). ER chaperones such as binding immunoglobulin protein (BiP) are Ca^2+^-binding proteins; thus, when Ca^2+^ is released from ER, these proteins are also secreted from ER, causing exodosis and activation of KDEL receptor (KDELR). Figure 2 is reproduced from figures in Aoe, T. Pathological Aspects of COVID-19 as a Conformational Disease and the Use of Pharmacological Chaperones as a Potential Therapeutic Strategy. *Front Pharmacol* 2020, 11, 1095 (doi:10.3389/fphar.2020.01095) [29].

Conversely, cytoplasmic calcium is transported into the ER by sarcoplasmic reticulum calcium ATPase (SERCA) on the ER membrane [44]. Changes in calcium homeostasis can affect protein folding in the ER, triggering the UPR as a rescue mechanism [35]. ER-resident proteins are often Ca^2+^-binding proteins and are involved in functions such as protein folding, modification, and transport. Under certain stress conditions, such as Ca^2+^ depletion in the ER lumen by thapsigargin treatment that inhibits SERCA, these ER proteins can undergo “exodosis”, in which they are released from the ER and even from the cells [45]. This protein efflux is a biomarker of ER Ca^2+^ depletion and indicates changes in cellular homeostasis [46]. Ca^2+^ release from the ER caused by the activation of cell surface receptors through CICR also induces exodosis and the depletion of ER molecular chaperones from the ER, which disturbs the folding environment in the ER and activates the UPR [35] (Figure 2).

Coordination between UPR signaling and energy demands occurs in the mitochondria-associated membrane [47], which is a subdomain specialized for inter-organelle communication [35]. Ca^2+^ uptake in the mitochondria is controlled by the mitochondrial calcium uniporter (MCU) complex, which is composed of several subunits [48]. Changes in cytoplasmic Ca^2+^ levels induce conformational changes in MCU regulatory proteins and stimulate channel activity [48]. ER Ca^2+^ release influences the mitochondrial metabolism and fine-tunes the threshold for apoptosis under chronic stress conditions [35].

### 3.3. UPR

When the ER faces stress, such as ischemia, hypoxia, toxic substances, or increased protein production, the resulting accumulation of misfolded proteins activates the UPR [2]. The UPR restores normal cellular function by suppressing protein translation, promoting the degradation of misfolded proteins, and facilitating the production of molecular chaperones [2,28]. The UPR involves three main stress kinases: inositol-requiring enzyme 1α (IRE1α), protein kinase R (PKR)-like ER kinase (PERK), and activating transcription factor 6 (ATF6) [2]. ATF6 is a type II transmembrane protein that resides in the ER, but upon ER stress, it moves from the ER to the Golgi apparatus, where it is cleaved [49]. This processing involves dissociation from BiP and the exposure of Golgi localization signals [50]. The cleaved ATF6 fragment translocates to the nucleus as a transcription factor and promotes the transcription of genes encoding ER chaperones and components of ER-associated degradation (ERAD) [49]. PERK is another ER stress sensor kinase. Upon ER stress, PERK activates eukaryotic initiation factor 2α (eIF2α), leading to a reduction in general protein synthesis, which helps alleviate the load of unfolded proteins in the ER [51]. PERK also upregulates activating transcription factor 4 (ATF4), which is a transcription factor that activates genes involved in the UPR, amino acid metabolism, and redox reactions [52,53]. The activation of PERK induces the expression of the transcription factor C/EBP homologous protein (CHOP) and promotes apoptosis under prolonged ER stress [54,55]. IRE1α is an ER-resident kinase and endoribonuclease. Upon activation, IRE1α splices *X-box binding protein 1* (*XBP1*) mRNA, producing a potent transcription factor. The spliced *XBP1* drives the transcription of UPR target genes related to protein folding and ERAD, which are essential for restoring ER function [56]. IRE1α signaling can lead to adaptation or cell death, depending on the extent of ER stress. Under acute stress, IRE1α facilitates cell survival by enhancing the folding capacity in the ER [57]. IRE1α RNAase also degrades mRNAs other than *XBP1* by regulated IRE1-dependent mRNA decay (RIDD) [58]. In chronic ER stress conditions, IRE1α can promote cell death through RIDD [59]. In addition, IRE1α activates mitogen-activated protein (MAP) kinases, like c-jun amino-terminal kinase (JNK) [60], leading to inflammation [61]. Chronic ER stress, such as obesity, causes persistent inflammation in adipocytes and macrophages and induces the increased expression of the proinflammatory cytokines interleukin-6 (IL-6) and tumor necrosis factor alpha (TNFα) [62]. The modulation of ER stress by chemical chaperones suppresses nuclear factor-kappa B (NF-κB) activity and inflammation in mice with diet-induced obesity [63].

These ER stress kinases (IRE1α, PERK, and ATF6) are bound to BiP at rest, and are activated when BiP dissociates from these kinases and binds to the increased misfolded proteins under ER stress [64]. Therefore, BiP assists in the protein folding of newly synthesized polypeptides, the sensing of misfolded proteins, and the initiation of the UPR to restore ER homeostasis [31,65,66].

### 3.4. The KDEL Receptor and Exodosis

ER chaperones such as BiP and calreticulin are localized in the ER and maintain protein folding [31]. These molecular chaperones are Ca^2+^-binding proteins that are retained in the ER, although some are inadvertently leaked from the ER by coat protein II (COPII) vesicles [67]. These chaperones have a Lys-Asp-Glu-Leu (KDEL) amino acid sequence at their carboxy terminus, which is recognized by the KDEL receptor in the Golgi apparatus [68]. Subsequently, the chaperones are transported back to the ER by coat protein I (COPI) vesicles [69]. Misfolded proteins bound to ER molecular chaperones are also secreted from the ER and transported back to the ER by the KDEL receptor [70]. The KDEL receptor regulates COPI vesicular transport and contributes to protein quality control in the ER [34]. There are three genes for the human KDEL receptor—*KDELR1*, *KDELR2*, and *KDELR3*—and the transcription of *KDELR2* is increased by the UPR [67]. The UPR increases the production of ER molecular chaperones like BiP and also increases KDELR2 expression. Therefore, the recovery of ER molecular chaperones bound to misfolded proteins secreted from the ER also increases [70]. However, when more misfolded proteins accumulate or when Ca^2+^ is rapidly released from the ER, the ER chaperones exceeding the retrieval capacity of the KDEL receptors are secreted from the Golgi and then further secreted extracellularly. The process of the secretion of ER-resident proteins from the Golgi and to outside the cell is termed exodosis [45]. The KDEL receptor is a seven-transmembrane GTP-coupled receptor that, like cell surface receptors such as the mu opioid receptor, is activated by a ligand—an ER chaperone with a KDEL sequence [34]. Activation of the KDEL receptor activates protein kinase A [71] and ADP ribosylation factor GTPase activating protein 1 (ARFGAP1), promoting COPI reverse transport [69]. In addition, the activation of MAP kinase [72] and Src kinase [73] has been observed, and these kinases are thought to constitute part of the UPR reaction [74] (Figure 2).

## 4. ER Stress and Pain

**Figure 3:**
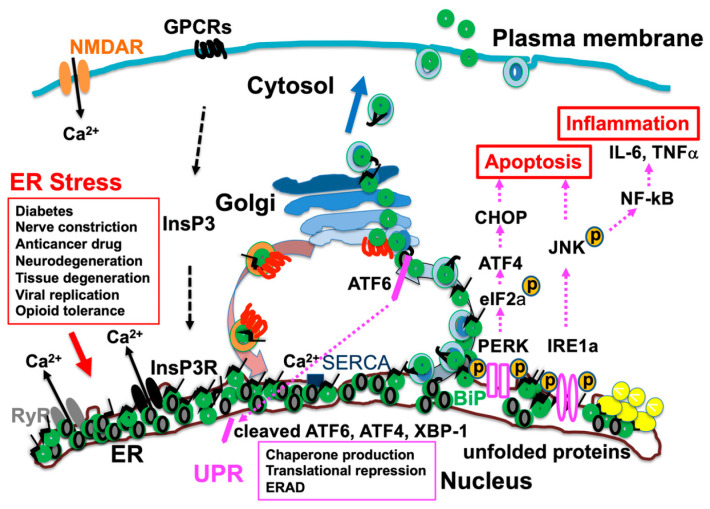
Accumulation of misfolded proteins due to endoplasmic reticulum (ER) stress results in binding immunoglobulin protein (BiP) dissociating from activating transcription factor 6 (ATF6), protein kinase R (PKR)-like ER kinase (PERK), and inositol-requiring enzyme 1α (IRE1α) and binding to misfolded proteins. Consequently, ATF6, PERK, and IRE1α are activated, and unfolded protein response (UPR) causes translational repression and chaperone production and enhances ER-associated degradation (ERAD). Furthermore, activation of c-jun amino-terminal kinase (JNK) and transcription factor C/EBP homologous protein (CHOP) induces inflammatory responses, production of cytokines such as interleukin-6 (IL-6) and tumor necrosis factor alpha (TNFα), and cell death. Various pathological conditions cause cell damage through ER stress and UPR, leading to pain. NMDAR, N-methyl-D-aspartate receptor; GPCRs, G protein-coupled receptors; NF-κB, nuclear factor-kappa B; SERCA, sarcoplasmic reticulum calcium ATPase; InsP3, inositol 1,4,5-trisphosphate; InsP3R, inositol 1,4,5-trisphosphate receptor; RyR, ryanodine receptor. Figure 3 is reproduced from figures in Aoe, T. Pathological Aspects of COVID-19 as a Conformational Disease and the Use of Pharmacological Chaperones as a Potential Therapeutic Strategy. *Front Pharmacol* 2020, 11, 1095 (doi:10.3389/fphar.2020.01095) [29].

ER stress has attracted attention as an important factor in the induction of pain. Recent research is uncovering the complex relationship between pain and the ER stress response, emphasizing how this connection may offer novel approaches for pain management [75] (Figure 3).

### 4.1. ER Stress in Pain Induction

ER stress has an important role in the induction of pain, particularly in neuropathic pain conditions [11,12,76]. Neuropathic pain, often resulting from nerve damage or a malfunctioning nervous system, is notoriously difficult to treat [12,76,77]. The role of ER stress in pain is not limited to neuropathic pain but extends to various medical conditions, including diabetes-induced neuropathic pain, inflammatory pain, and pain associated with diseases like osteoarthritis and viral infection. The ER stress response varies across these conditions, providing insights into the development of condition-specific pain management strategies.

#### 4.1.1. ER Stress in Spinal Nerve and Neuropathic Pain

Spinal nerve ligation (SNL) in rats induced the overexpression of ER stress markers, such as BiP and spliced *XBP1*, in spinal dorsal horn neurons [11]. The administration of tauroursodeoxycholic acid (TUDCA), which is an ER chemical chaperone that assists in protein folding [29], relieved ER stress and nociceptive behavior in SNL rats. In addition, ER stress in spinal dorsal horn neurons induced by the intrathecal injection of thapsigargin (which disturbs calcium homeostasis in the ER) in healthy rats resulted in mechanical hyperalgesia [78]. These studies indicated that ER stress is involved in neuropathic pain.

#### 4.1.2. Diabetic Neuropathy

Recent research indicates that ER stress is a significant cause of the development of diabetic peripheral neuropathy, which is a common complication of diabetes and is characterized by chronic pain or loss of sensation [79]. In diabetic neuropathy, ER stress markers are upregulated. However, these elevated markers of ER stress and neuralgia can be reversed by chemical chaperones, indicating a direct link between ER stress and pain induction in this condition [12]. In addition, chemical inducers of ER stress, such as tunicamycin, consistently induce pain behavior in healthy animals [12]. The role of protein kinase C epsilon (PKCε) in mediating chronic pain through ER stress and autophagy in diabetic neuropathy has been reported [13]. In a mouse model of diabetic neuropathic pain, PKCε upregulation was correlated with the increased expression of ER-stress-related molecules and autophagosome formation. Moreover, a PKCε-specific inhibitor was shown to suppress ER stress and autophagosome formation and reduce neuropathic pain [13].

#### 4.1.3. Postherpetic Neuralgia

Postherpetic neuralgia (PHN) is a common complication of herpes zoster and is challenging to treat [80]. In a study performed in the United Kingdom, 13.7% of herpes zoster patients complained of PHN pain 3 months after their diagnosis [81]. P2X7 receptors, which are cell surface receptors with ATP as a ligand, are ligand-gated, non-selective cation channels for Na^+^, Ca^2+^, and K^+^ [82]. Research in a rat PHN model suggested that the P2X7 receptor antagonist, brilliant blue G, inhibits ER stress and pyroptosis and increases pain thresholds, thus alleviating PHN [83]. This finding highlights ER stress as a key player in PHN pathophysiology.

#### 4.1.4. Pain-Related Central Nerve Lesions

Glial cells in perithalamic lesions of central post-stroke-pain model rats had decreased expression of soluble epoxy hydrolase, which was associated with increased expression of ER stress markers [77]. Furthermore, the increased phosphorylation of inflammatory MAP kinases (p38 and JNK) and the activation of glial cells were observed. The intrathalamic injection of soluble epoxy hydrolase increased the paw withdrawal mechanical threshold, indicating analgesic effects, and decreased ER stress and MAPK signaling in microglia and astrocytes around the lesion [77].

Increased levels of inflammatory cells and ER stress markers have been observed in the postmortem dorsal root ganglia of multiple sclerosis patients [84]. An experimental autoimmune encephalomyelitis mouse model of multiple sclerosis revealed that ER stress in the dorsal root ganglia regulated large-conductance potassium channels, contributing to pain [84]. The administration of the chemical chaperone 4-phenylbutyric acid (4-PBA) reduced ER stress as well as pain hypersensitivity in the mice.

In Parkinson’s disease, intracellular aggregates called Lewy bodies are observed [85]. Protein degradation in the proteasome is inhibited, and dopaminergic neurons are damaged by ER stress [10,86]. In addition to movement disorders, the patients often experience pain [87]. Lewy bodies are also found in their spinal cords and may cause neuropathic pain [88].

#### 4.1.5. Chemotherapy-Induced Peripheral Neuropathy

Chemotherapy for cancer patients often causes peripheral neuropathy [89]. Although oxidative stress contributes to the pathology of chemotherapy-induced peripheral neuropathy (CIPN) [90], ER stress is also involved through anticancer drugs such as vincristine and paclitaxel [91,92]. Phytochemicals that alleviate ER stress have been suggested to be useful in preventing CIPN [76,93,94].

#### 4.1.6. Opioid-Induced Hyperalgesia and Tolerance

Taking opioids for a long time can cause hyperalgesia and tolerance, which reduce the analgesic effect of the drugs [95]. Morphine induces ER stress by activating the mu opioid receptor, leading to defective autophagy and subsequent astrocyte activation [96]. ER stress has been linked to opioid-induced hyperalgesia (OIH). Morphine activates the three arms of the UPR: IRE1α/XBP1, protein kinase PERK/eIF2α, and ATF6. Among these, the inhibition of IRE1α/XBP1 or ATF6 attenuated the development of OIH in a rodent model [14]. Furthermore, the administration of ER chemical chaperones such as 4-PBA and TUDCA suppressed the development of opioid tolerance and preserved analgesic effects in a mouse model [97]. Therefore, modulation of the ER stress response presents a novel treatment strategy for OIH and opioid tolerance.

#### 4.1.7. ER Stress in Inflammatory Pain

The involvement of ER stress has also been observed in nociceptive pain models that do not directly damage nerves. In a rat model of orofacial inflammatory pain induced by injection with complete Freund’s adjuvant (CFA), thermal pain hypersensitivity was significantly increased [15]. In addition, BiP and p-eIF2α expression were significantly increased in the trigeminal ganglion of these rats. However, salubrinal, which reduces ER stress, attenuated the CFA-induced hypersensitivity to heat pain [15]. In another study, formalin injection into the plantar surface of rats significantly upregulated ER stress markers and c-fos in the spinal cord. This upregulation and nociceptive behavior were reduced by peritoneal injection of the chemical chaperone 4-PBA [98].

#### 4.1.8. ER Stress in Intervertebral Disc Degeneration and Osteoarthritis

Intervertebral disc degeneration and osteoarthritis (OA) are major causes of chronic pain. Secretory cells such as chondrocytes are highly sensitive to ER stress [99]; consequently, ER stress influences cartilage formation and degeneration, and osteoarthritis. ER stress is also implicated in inflammation, cellular senescence, apoptosis, and extracellular matrix dysregulation in intervertebral disc degeneration [100]. The potential of naturally derived ER stress inhibitors in slowing the progression of OA has been highlighted [101]. In addition, targeting chondrocyte ER stress has emerged as a novel therapeutic approach [102].

The studies cited above consistently highlight the crucial role of ER stress in the development and maintenance of various pain states, especially neuropathic pain. Modulation of the ER stress pathways presents a promising therapeutic target for pain management. Further research in this domain may pave the way for novel, effective treatments for chronic pain conditions.

### 4.2. Molecular Pathways between Pain and ER Stress

#### 4.2.1. Neurotoxicity by ER Stress

As previously stated, pathological conditions such as ischemia, hypoxia, toxic substances, and oxidative stress may disturb protein folding in the ER. An accumulation of misfolded proteins in the ER leads to ER stress and the UPR. Uncompensated ER stress causes aberrant intracellular signaling, cellular dysfunction, and cell death [2]. When this process happens to sensory neurons, neuropathic pain may occur [12,78]. Neuroinflammation caused by viral infection or autoimmune disease [103] and oxidative stress associated with inflammation caused by trauma or physical constrictions may cause neuropathic pain [11,83,100]. In addition, neurotoxic drugs such as anticancer drugs might impair protein folding in the ER through oxidative stress, resulting in ER stress [76]. In shingles, herpes zoster virus proteins are produced within infected sensory neurons. The explosive production of these foreign gene-derived heterologous proteins in the ER places considerable stress on the folding environment, causing cell damage and cell death [104], which results in pain sensations or numbness [83]. The intense production of foreign proteins in sensory neurons through other viral infections or mRNA vaccines, such as the one against severe acute respiratory syndrome coronavirus-2 (SARS-CoV-2), could induce similar pathologies [29]. Neurodegenerative diseases such as Parkinson’s disease and Huntington’s disease are thought to exhibit neuronal damage due to ER stress caused by intracellular protein aggregation [10,86,105,106]. Although the main symptoms of these diseases are movement disorders, it could be speculated that neuropathic pain may occur if sensory neurons or nerve cells in brain areas that control pain sensations are damaged. In fact, these patients often complain of pain [99,100].

In addition, crosstalk between UPR signaling and the signaling pathways that transmit painful stimuli may be perceived as a painful sensation. IRE1α–XBP1 activation induces the biosynthesis of prostaglandins, including the nociceptive mediator prostaglandin E2 (PGE2). Mice treated with an IRE1α inhibitor exhibited reduced pain behavior in a PGE2-dependent pain model [107]. Another study elucidated the role of PERK and IRE1 in regulating the expression of lipocalin-2 and nod-like receptor family pyrin domain containing 3 in astrocytes, contributing to morphine tolerance and hyperalgesia in a rodent model [108]. Pharmacological inhibition of PERK and IRE1 may be a possible therapeutic target for morphine tolerance and hyperalgesia.

#### 4.2.2. Changing Calcium Dynamics

ER stress is induced not only by disrupting protein folding, such as with tunicamycin, which inhibits N-linked glycosylation in the ER [109], but also by changing calcium dynamics, such as with thapsigargin, which disturbs SERCA2 in the ER [44]. In the rat chronic constriction injury neuropathic pain model, SERCA2b, which is the major SERCA isoform in the dorsal root ganglia, is decreased at the mRNA, protein, and activity levels [110]. Moreover, the inhibition of SERCA by thapsigargin causes ER stress, accompanied by neuronal hyperexcitability, nerve damage, satellite glial cell activation, and mechanical allodynia [78,110]. SERCA2b activators may therefore be potential therapeutic agents for neuropathic pain relief [110]. Such changes in calcium dynamics also occur following the activation of cell surface receptors by ligands. The mu opioid receptor is a GPCR, and the stimulation of this receptor by opioids such as fentanyl activates phospholipase C, producing InsP3 [37]. InsP3 then binds to receptors in the ER membrane, and calcium is released from the ER through the InsP3 receptor [111]. The repetition of these reactions decreases the Ca^2+^ concentration in the ER. In addition, NMDA receptors are involved in neuropathic pain, and their antagonists, like ketamine, exhibit analgesic effects [112]. NMDA receptors are ion channels and their activation causes a flow of cations such as Ca^2+^ and Na^+^ into cells, and through the process of CICR, Ca^2+^ is subsequently released from the ER into the cytoplasm through ryanodine receptors [39]. Therefore, Ca^2+^ is released from the ER upon the activation of cell surface receptors in various pathological conditions, including pain conditions.

Prolonged use of opioids causes opioid tolerance and hyperalgesia [95]. Although fentanyl and remifentanyl are used during surgery, opioid tolerance and hyperalgesia have been observed immediately after surgery [113]. Animal studies have shown that opioid tolerance and hyperalgesia can be alleviated by the combined use of chemical chaperones that compensate for the function of ER molecular chaperones [97]. Knock-in mice expressing mutant BiP lacking the KDEL sequence at the carboxy terminus are less likely to develop opioid tolerance, with the analgesic effect of opioids being maintained even with chronic use [114]. Mu opioid receptor activation causes IP3-mediated Ca^2+^ release from the ER. Thus, activation of the KDEL receptor through an exodus of ER molecular chaperones with the KDEL sequence may be involved in opioid tolerance and hyperalgesia. The UPR response, which includes the activation of KDEL receptors, may crosstalk with the pain information transmission system in sensory neurons through the activation of MAP kinase, JNK, PKA, Src kinase, and so on, and may affect pain transmission [34] (Figure 3).

### 4.3. Therapeutic Implications Based on Pain and ER Stress

The involvement of ER stress in pain suggests that targeting ER stress pathways could be a novel approach for pain management. Strategies using chemical chaperones to reduce ER stress and modulating UPR pathways have shown promise in preclinical studies.

#### 4.3.1. Chemical Chaperones and ER Stress Modulator

Chemical chaperones, such as 4-PBA [98,115] and TUDCA [78], facilitate protein folding, thereby reducing ER stress [29]. The efficacy of chemical chaperones has been demonstrated in conditions like diabetic peripheral neuropathy [115,116], formalin-induced pain [98], and the SNL rat model [78]. Salubrinal inhibits the dephosphorylation of eIF2α and protects cells from ER stress caused by protein overload [117]. Salubrinal also reduced hyperalgesia in the SNL rat model [118]. Several clinical trials of TUDCA [119] and 4-PBA [120] for ER-stress-related diseases like amyotrophic lateral sclerosis have been reported.

#### 4.3.2. Phytochemicals and Natural Compounds

Several natural compounds have demonstrated potential in ameliorating ER-stress-related pain [76] (Table 1). These compounds often exhibit multiple mechanisms of action, including antioxidant properties and ER stress modulation. Eucommia is an herb used in Traditional Chinese Medicine. Geniposide is an effective compound involved in the therapeutic effects of Eucommia on osteoporosis [121]. Geniposide ameliorated dexamethasone-induced ER stress and mitochondrial apoptosis in osteoblasts [122]. Asarone is a chemical compound found in several plants, such as those of the genus *Acorus*. In a rat model of chronic constriction injury-induced neuropathic pain, ER stress was induced in the spinal cord, and α-asarone alleviated ER stress and improved neuropathic pain [123]. In animal experiments, hesperidin and aucubin alleviated ER stress and neuropathic pain in CIPN [93,94].

#### 4.3.3. Conventional Drugs with ER-Stress-Modulating Effects

NSAIDs exhibit excellent analgesic effects against nociceptive pain through cyclooxygenase inhibition [124], but various effects related to ER stress have also been reported. The long-term administration of NSAIDs can lead to serious gastrointestinal complications such as ulceration, bleeding, and perforation [125,126]. Diclofenac and indomethacin were reported to induce apoptosis in cultured cells by promoting the upregulation of the pro-apoptotic PERK–eIF2α–CHOP pathway [127]. Diclofenac also decreased the expression of the pro-survival factor ATF6 [127]. Conversely, low concentrations of diclofenac suppressed the expression of the CHOP gene and cell death in endothelial cells treated with tunicamycin [128]. Increased CHOP expression has been observed with many NSAIDs, such as diclofenac, ibuprofen, and celecoxib [129], whereas chaperone activity has been reported with flurbiprofen, which decreases CHOP expression [130]. Thus, flurbiprofen reduces the accumulation of unfolded proteins, whereas celecoxib blocks ER Ca^2+^-ATPases [131] and causes ER stress [132].

A study on ketamine demonstrated the efficacy of this drug in reducing neuropathic pain in rats through modulating ER stress markers [112]. In a rat neuropathic pain model with sciatic nerve ligation, increased expressions of ER stress markers were observed. The systemic administration of ketamine reduced the expression of NMDA receptor subtype 2B and ATF-6, and also improved pain symptoms [112].

Autophagy following ER stress degrades excess ER via autophagy processing (ER-phagy) and maintains cellular homeostasis [133]. In SNL rats, ER stress and impaired ER-phagy were induced in the spinal cord, and dexmedetomidine produced analgesic effects through increased ER-phagy [134,135].

#### 4.3.4. Stem Cell Therapy

Stem cells from human exfoliated deciduous teeth were found to alleviate trigeminal neuralgia by reducing ER stress, offering a novel therapeutic avenue [136]. Exosomes derived from mesenchymal stem cells can modulate ER stress and inhibit apoptosis in nucleus pulposus cells. This modulation could potentially slow the progression of intervertebral disc degeneration [137].

#### 4.3.5. Current Limitations of ER Stress in Pain Therapy

The relationship between ER stress and pain is being shown through preclinical studies with animal experiments. However, there seem to be no clinical data in humans at present, which is a limitation of the research field and of this review article. Therefore, although targeting ER stress in pain therapy shows promise, challenges still remain. These challenges include the specificity of agents for pain-related ER stress, potential systemic side effects, and the translation of preclinical findings to effective clinical treatments. Future research should focus on refining these therapeutic strategies, understanding the complexities of ER stress in different pain conditions, and developing tailored treatments.

## 5. Conclusions

Elucidating the molecular pathways linking ER stress to pain and understanding the implications of modulating these pathways offer a promising therapeutic strategy for various pain conditions. From chemical chaperones to conventional drugs and stem cell therapy, the scope of potential treatments is wide and varied. As research progresses, these approaches could revolutionize pain management, offering relief to those experiencing chronic and debilitating pain conditions. Future research in this field is essential for developing effective pain therapies targeting ER stress.

## Figures and Tables

**Table 1:** Phytochemicals ameliorating ER-stress-related pain.

Phytochemical	Origin	Effects	Disease	Reference
Hesperidin	A dihydroflavone compound in citrus peel	Reduces ER stress and neuropathic pain	CIPN	[93]
Aucubin	An iridoid glycoside found in asterids such as Aucuba japonica	Reduces ER stress and neuropathic pain	CIPN	[94]
Geniposide	An iridoid glucoside extracted from Eucommia	Reduces ER stress and apoptosis	Osteoporosis	[122]
α-Asarone	A chemical compound of the phenylpropanoid class found in the genus Acorus	Reduces ER stress and neuropathic pain	Chronic constriction injury-induced neuropathic pain	[123]

CIPN, chemotherapy-induced peripheral neuropathy.

## Abbreviations

ER	endoplasmic reticulum
UPR	unfolded protein response
COPII	coat protein II
COPI	coat protein I
GPCRs	G protein-coupled receptors
MOR	μ-opioid receptor
InsP3	inositol 1,4,5-trisphosphate
CICR	calcium-induced calcium release
NMDAR	N-methyl-D-aspartate
SERCA	sarcoplasmic reticulum calcium ATPase
IRE1α	inositol-requiring enzyme 1α
PERK	protein kinase R (PKR)-like ER kinase
ATF6	activating transcription factor 6
ERAD	ER-associated degradation
eIF2α	eukaryotic initiation factor 2α
ATF4	activating transcription factor 4
CHOP	C/EBP homologous protein
XBP1	X-box binding protein 1
RIDD	regulated IRE1-dependent mRNA decay
MAP	mitogen-activated protein
JNKs	c-Jun amino-terminal kinases
IL-6	interleukin-6
TNFα	tumor necrosis factor alpha
NF-κB	nuclear factor-kappa B
ARFGAP1	ADP ribosylation factor GTPase activating protein 1
SNL	spinal nerve ligation
TUDCA	tauroursodeoxycholic acid
PKCε	protein kinase C epsilon
PHN	postherpetic neuralgia
4-PBA	4-phenylbutyric acid
CIPN	chemotherapy-induced peripheral neuropathy
OIH	opioid-induced hyperalgesia
CFA	complete Freund’s adjuvant
OA	osteoarthritis
SARS-CoV-2	severe acute respiratory syndrome coronavirus-2
PKA	protein kinase A
